# Modeling the functional state of the reverse transcriptase of hepatitis B virus and its application to probing drug-protein interaction

**DOI:** 10.1186/s12859-016-1116-4

**Published:** 2016-08-31

**Authors:** Xiaojun Xu, Hong Thai, Kathryn M. Kitrinos, Guoliang Xia, Anuj Gaggar, Matthew Paulson, Lilia Ganova-Raeva, Yury Khudyakov, James Lara

**Affiliations:** 1 Division of Viral Hepatitis, National Center for HIV, Hepatitis, STD and TB Prevention, Centers for Disease Control and Prevention, Atlanta, GA 30333 USA; 2 Gilead Sciences, Inc., Foster City, CA USA

**Keywords:** Hybrid structure modeling, Molecular dynamics (MD), Solvated interaction energy (SIE), Hepatitis B, Tenofovir, Drug resistance, Reverse transcriptase

## Abstract

**Background:**

Herein, the predicted atomic structures of five representative sequence variants of the reverse transcriptase protein (RT) of hepatitis B virus (HBV), sampled from patients with rapid or slow response to tenofovir disoproxil fumarate (TDF) treatment, have been examined to identify structural variations between them in order to assess structural and functional properties of HBV-RT variants associated with the differential responses to TDF treatment.

**Results:**

We utilized a hybrid computational approach to model the atomistic structures of HBV-RT/DNA-RNA/dATP and HBV-RT/DNA-RNA/TFV-DP (tenofovir diphosphate) complexes with the native hybrid DNA-RNA substrate in place. Multi-nanosecond molecular dynamics (MD) simulations of HBV-RT/DNA-RNA/dATP complexes revealed strong coupling of the natural nucleotide substrate, dATP, to the active site of the RT, and the differential involvement of the two putative magnesium cations (Mg^2+^) at the active site, whereby one Mg^2+^ directly bridges the interaction between dATP and HBV-RT and the other serves as a coordinator to maintain an optimal configuration of the active site. Solvated interaction energy (SIE) calculated in MD simulations of HBV-RT/DNA-RNA/TFV-DP complexes indicate no differential binding affinity between TFV-DP and HBV-RT variants identified in patients with slow or rapid response to TDF treatment.

**Conclusion:**

The predicted atomic structures accurately represent functional states of HBV-RT. The equivalent interaction between TFV-DP and each examined HBV-RT variants suggests that binding affinity of TFV-DP to HBV-RT is not associated with delayed viral clearance.

**Electronic supplementary material:**

The online version of this article (doi:10.1186/s12859-016-1116-4) contains supplementary material, which is available to authorized users.

## Background

During the life cycle of HBV, RT utilizes a single-stranded viral genomic RNA as a template to synthesize a hybrid RNA-DNA duplex, and then converts it to double-stranded DNA (ds-DNA). As this step is critical for the viral genome replication, it makes RT an attractive target for antiviral treatment. Thus, both nucleoside and nucleotide RT inhibitors (NRTIs) and non-nucleoside RT inhibitors (NNRTI) are extensively used as antiretroviral agents against HBV and HIV infection [1, 2].

**Fig. 1:**
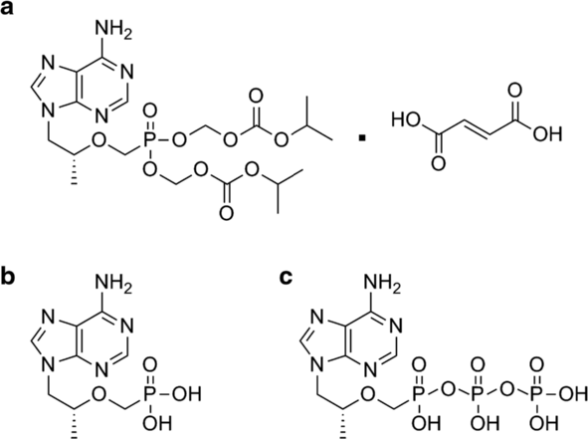
*Chemical structures of* **a** tenofovir disoproxil fumarate (TDF), prodrug of **b** tenofovir (TFV); **c** tenofovir diphosphate (TFV-DP)

The effective HBV NRTIs are nucleoside or nucleotide analogs that can be phosphorylated to their diphosphate or triphosphate active forms by intracellular kinases. Active forms of NRTIs incorporate into the elongating DNA strand, terminate the elongation of the nascent DNA strand, and prevent additional dNTPs from being incorporated. Tenofovir disoproxil fumarate (TDF) is an oral prodrug of nucleotide analog tenofovir (TFV, PMPA, 9-[(R)-2-(phosphonomethoxy)propyl]adenine). TDF is rapidly converted to TFV following absorption and is readily catalyzed to the active diphosphate form, TFV-DP (Fig. 1) [3].

TDF is a non-selective RT inhibitor with demonstrated in vitro activity against both wild-type and mutant strains of HIV [4–6] and is also effective for treating chronic hepatitis B (CHB) [7–10]. While resistance mutations within the HBV-RT have been identified for NRTI monotherapies, such as adefovir, entecavir, lamivudine, telbivudine and clevudine [8], there is no evidence of TDF resistance [11]. However, differential therapy responses can be observed among patients and have been previously defined: a delayed viral clearance response in slow responders (SR), or a rapid decline in viral load immediately following treatment initiation in rapid responders (RR) [12, 13].

TFV resistance in the setting of HIV has been extensively studied at the molecular level [14–16]. These studies have shown that the molecular mechanism of TFV resistance is complex and involves reverse transcriptase, RNase and exonuclease activities of HIV-RT. In order to remain functional, resistant mutants of HIV-RT need to maintain their capability of incorporating nucleotide at a sufficient rate, as well as to discriminate against NRTIs or improve the excision rate of incorporated NRTIs [17].

As the HBV-RT is homologous to HIV-RT [17], it is reasonable to propose that a similar mechanism could be involved in the differential treatment response to TDF. Whether and/or how the molecular level interaction between TFV-DP and HBV-RT has a part in the aforementioned differential patient responses upon TDF treatment is unclear due to the lack of direct experimental evidence. There is no solved structure of the HBV-RT in any form.

In this study, we set out to address the molecular level interaction between TFV-DP and HBV-RT and the relationship to patient response using a hybrid computational approach. We utilized two X-ray crystal structures of HIV-RT to model the atomic structure of HBV-RT in its functional state with the aim of delineating the detailed drug-protein interactions between TFV-DP and the active sites of genetically distinct variants of HBV-RT.

## Results and discussion

### Atomic structure models of the HBV-RT in its functional state

While substantial efforts have been devoted to solving the crystal structures of HIV-RT, with or without ligands (e.g. substrates, small inhibitory molecules), no experimentally derived HBV-RT structures have become available. HIV-RT and HBV-RT are recognized as homologous [17], and possess similar multiple catalytic activities. A computational approach is often applied to structural implications in drug-resistance studies on HBV-RT using HIV-RT as structural template for homology modeling of HBV-RT [17, 18]. Still, the low sequence identity and modeling HBV RT without considering the particular functional state imposes technical difficulties in obtaining structural models of high confidence.

In models of NRTIs bound to HBV-RT, it is reasonable to hypothesize that an effective drug binds to the active site as if it were a native substrate in position to be further covalently incorporated, similar to the HIV-RT/DNA-DNA/TFV-DP complex [19]. Even though we are not to rule out the possibility of a NRTI being able to bind elsewhere than the active site on HBV-RT or HIV-RT, to date there is no evidence for such type of interaction. Moreover, a putative strong binding of the drug to the active site would seem to preclude the likelihood of additional binding sites on the enzyme. To use constraints derived from experimental results to guide the refinement, we first modeled the HBV-RT structure itself using ITASSER [20, 21] with an additional template, the X-ray crystal structure of HIV-RT/DNA-RNA/dATP [22], and then included the native substrates (the DNA-RNA duplex and dATP) from this template complex in the subsequent refinement (MD simulation).

The C-scores [20] (data not shown) of our HBV-RT models generated by ITASSER for the variants of interest indicated high confidence; however, we noticed that the exact folding patterns in the fingers domain differ (Additional file 1: Figure S1). Therefore, we chose the HBV-RT4 model (HBV-RT4, Additional file 1: Figure S1C) which has the same folding pattern as the HIV-RT template (Additional file 1: Figure S1F) in order to proceed with the refinement.

The structure of the N-terminal 40 amino acid region was of relatively low accuracy (Additional file 1: Figure S1A-E). Thus, we re-modeled the N-terminal of HBV-RT4 separately using ITASSER, and assembled it with the rest of the protein using *ab initio* assembly [23]. The N-terminus in the resultant assembled structure of HBV-RT4 is of much more ordered secondary structural elements than it was in the initial model from ITASSER (Additional file 2: Figure S2).

The assembled structure was then combined with the duplex DNA-RNA and dATP from the template HIV-RT/DNA-RNA/dATP crystal strcuture, refined by ~120 ns MD simulation in explicit solvent to obtain the final model of HBV-RT4/DNA-RNA/dATP.

**Fig. 2:**
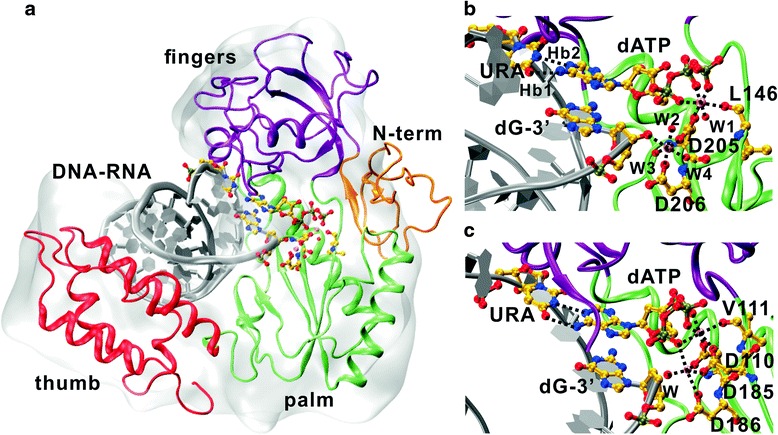
*The structure of HBV-RT4/DNA-RNA/dATP and the active site Mg* ^*2+*^ *network in comparison with that from the HIV-RT/DNA-RNA/dATP crystal structure.* **a** The HBV-RT4/DNA-RNA/dATP structure model is shown in cartoon with each domain in different color (N-terminal: orange; fingers: purple; thumb: red; palm: green; dsDNA: silver). The substrate dATP and the residues involved in direct interaction with it are in stick and ball representation. The corresponding part from the template HIV-RT/DNA-RNA/dATP (PDB code: 4PQU; chain A, residue 1 to residue 320) is shown in gray surface representation, structurally aligned to the HBV-RT4/DNA-RNA/dATP model; **b** The active site configuration of the HBV-RT4/DNA-RNA/dATP model. The non-bonded interactions are explicitly represented as black dashed lines as the two H-bonds (Hb1 and Hb2), and the ionic bonds formed between the Mg^2+^ ions (in red ball representation without labeling) and the chelating oxygen atoms. The water oxygen atoms participating in the chelation are labeled as from W1 to W4; **c** The active site configuration of the HIV-RT/DNA-RNA/dATP crystal structure, only one water molecule (labeled as W) is involved in the Mg^2+^ network. The D110 has no counterpart in the HBV-RT model. The hydrogen atoms are hided in the representations for clearance

After the MD simulation, the centroid of the dominant cluster (obtained by pairwise root mean square deviation, rmsd, clustering) from the second half of HBV-RT4/DNA-RNA/dATP trajectory was chosen as the representative structure for HBV-RT in its functional state (Additional file 2: Figure S2A). The corresponding part from the HIV-RT/DNA-RNA/dATP (HIV-RT residue 1 to 320) is shown in gray surface representation for comparison. Apart from the N-terminal domain partially protruding out of the template’s surface envelope, all the other domains (“thumb”, “fingers” and “palm” domains) are in close agreement with the template structure (Fig. 2a). The DNA-RNA duplex is clamped by the enzyme through the circular arrangement of the thumb, palm and fingers domains. Prior to entering the active site, the duplex is anchored by one α-helix (D283 to A297) in the thumb domain. The RNA template strand is tracked along by a long loop (N123 to N131) connecting the palm and fingers domains, and captured by the fingers domain toward its 5’ end.

In preparation for incorporation, the dATP situates itself in the active site with good base ring stacking to the penultimate dG, and by making two persistent hydrogen bonds (H-bond) with the uracil from the opposing template RNA strand (Fig. 2a, Additional file 2: Figure S2A); these features persist throughout the un-constrained MD (20 -120 ns). The triphosphate group of the dATP is stably coupled with residue L146 and D205 of HBV-RT by cooperatively chelating one Mg^2+^ (Fig. 2b). This particular configuration differs from the HIV-RT active site mainly by the absence of a third aspartate residue in the active site; in HIV-RT this third aspartate (D110) participates in chelating the two Mg^2+^ in the active site of the HIV-RT (Fig. 2c). Without this aspartate, the Mg^2+^ ions were stabilized over the equilibration stage of the MD by incorporating extra water molecules, and this constellation remained stable throughout in the production run (see next section).

### Different roles of Mg^2+^ ions in the active site

**Fig. 3:**
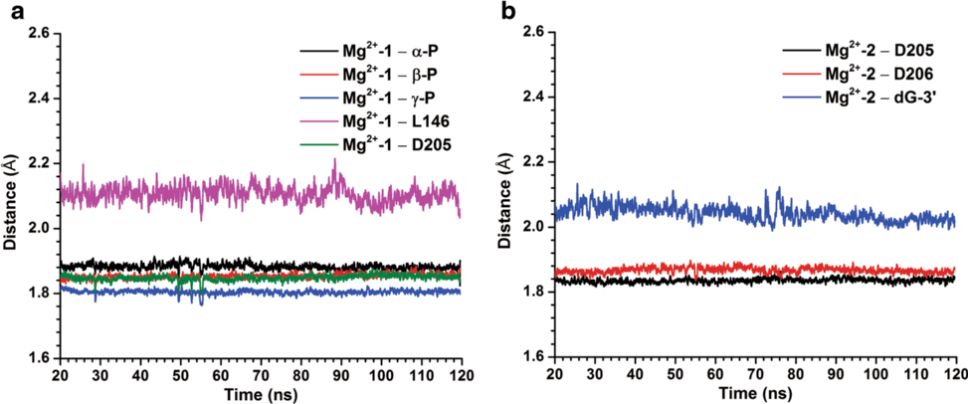
*Distances from the Mg* ^*2+*^ *ions to non-water atoms which participating in its chelation.* **a** The distance profile of the Mg^2+^-1 to the chelating atoms; **b** The distance profile of the Mg^2+^-2 to the chelating atoms. Each oxygen participating in the chelation is represented by the residue name

Apart from the first Mg^2+^ (Mg^2+^-1 hereafter) being directly involved in the dATP binding to the active site, a second Mg^2+^ (Mg^2+^-2 hereafter) is also present in the HIV-RT/DNA-RNA/dATP crystal structure. Due to an insufficiency of potential ligation atoms in the active site of HBV-RT comparing to HIV-RT, Mg^2+^-1 can only be coordinated by five surrounding oxygens and Mg^2+^-2 by only three, not considering contributions from water molecules postulated in our initial settings. In fact, water molecules filled in dynamically over the equilibration stage and stabilized the chelation as with the optimal octahedral configuration for Mg^2+^ over the entire MD simulation. To evaluate the stability of the chelation, we computed the moving average (0.2 ns window) of the distance from each non-water oxygen atom to the corresponding Mg^2+^ over the non-constrained MD simulation (20 – 120 ns). The result shows a persistently stable coupling of the non-water atoms to the corresponding Mg^2+^ ion (Fig. 3).

While it is clear that the Mg^2+^-1 bridges the interaction between the dATP and RT (present in the HIV-RT/DNA-RNA/dATP and HIV-RT/DNA-DNA/TFV-DP crystal structure [19, 22], PDB code: 4PQU and 1 T05), the importance of the Mg^2+^-2 in these RTs is open to question, as it is only Mg^2+^-1 which appears in the HIV-RT/DNA-DNA/TFV-DP structure (PDB code: 1 T05) [19]. To address this question, MD simulations with only the Mg^2+^-1 (i.e. by deleting the Mg^2+^-2 atom) were also performed for all the HBV-RT variants. On one hand, we found no significant changes in binding affinities of dATP to HBV-RT upon removal of Mg^2+^-2 (Table 1), which is consistent with the observation that the Mg^2+^-2 is not directly involved in the substrate binding. On the other hand, it is noticeable that oxygen from the hydroxyl group of dG-3’ is directly involved in the chelation of the Mg^2+^-2 throughout the simulation (Fig. 3b). Since the nature of the incorporation of a dNTP is to form a phosphodiester bond between the oxygen of the 3’-OH and the α-P of the triphosphate group of the dNTP, the network formed by the two Mg^2+^ ions is the optimal configuration for the impending chemical reaction. The distance between the oxygen of the 3’-OH and the α-P of the triphosphate group of the dATP was also monitored for each of the HBV-RT variant over the simulations (data not shown). As further separation of the 3’-OH and the α-P did not appear in any of our Mg^2+^-1 only system, the Mg^2+^ network seems to contribute to the function of the enzyme in a way other than merely maintaining the distance between the 3’-OH of the extending DNA strand and the α-P of the dATP. Presumably, forming a coordination bond with the Mg^2+^-2 would render the oxygen atom in the 3’-OH more favorable to initiation of the nucleophilic attack on the α-P, thereby flavoring the transcriptase activity of the enzyme.

**Table 1:** Approximated binding free energies with standard errors of dATP or TNV to HBV-RT/DNA-RNA using Solvated Interaction Energy method (SIE)

Category	Variant	dATP, one Mg^2+^ (Kcal mol^-1^)	dATP, two Mg^2+^ (Kcal mol^-1^)	TFV-DP, two Mg^2+^ (Kcal mol^-1^)
RR	RT5	−10.43 ± 0.02	−9.93 ± 0.02	−8.91 ± 0.01
	RT3	−11.51 ± 0.02	−11.21 ± 0.03	−9.22 ± 0.01
SR	RT1	−10.59 ± 0.01	−9.69 ± 0.02	−9.56 ± 0.01
	RT4	−12.51 ± 0.01	−9.50 ± 0.02	−9.05 ± 0.02
	RT2	−10.51 ± 0.02	−10.59 ± 0.01	−9.51 ± 0.01

### Binding free energy of TFV-DP to HBV-RT

Drug resistance often arises from structural alterations consequent to mutations in the target protein, which somehow disrupts or interferes with drug binding. To understand the underlying mechanism of the differential response to TDF treatment, we start by asking whether the TFV-DP binds differently to the RT variants from patients with slow or rapid response to TDF. In building a structural model of HBV-RT4/DNA-RNA/TFV-DP, we started with the HBV-RT4 model generated by ITASSER and AIDA (Additional file 2: Figure S2), then inserted the duplex DNA-RNA taken originally from the HIV-RT/DNA-RNA/dATP template structure [22], and finally incorporated the TFV-DP from the HIV-RT/DNA-DNA/TFV-DP structure (PDB code: 1 T05) [19]. Models for the other four variants were built analogously from the homology models we obtained using the HBV-RT4 as template, into which the same DNA-RNA duplex and TFV-DP were incorporated. We then pursued MD simulations to refine all the HBV-RT/DNA-RNA/TFV-DP structures. The centroid of the major cluster from the second half of each MD trajectory was then chosen as representative model.

**Fig. 4:**
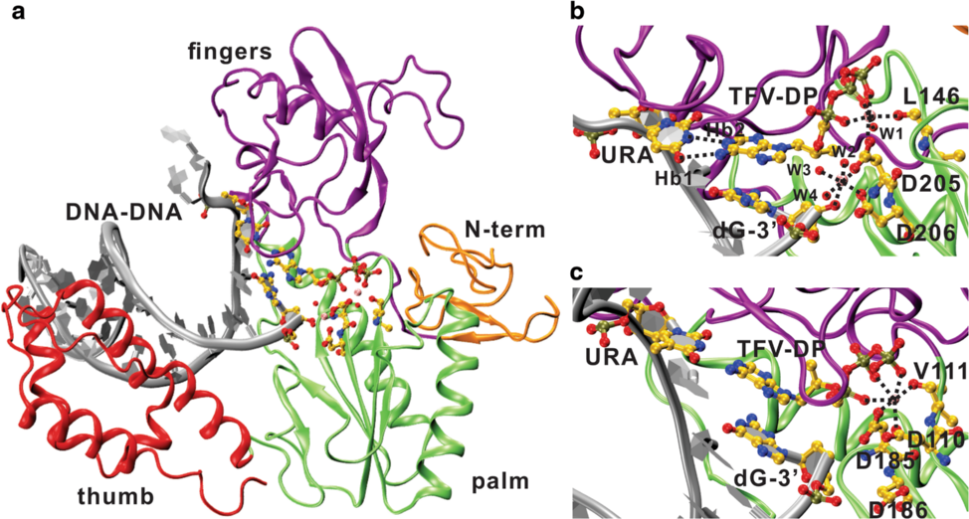
*The structure of HBV-RT4/DNA-RNA/TFV-DP and the active site Mg* ^*2+*^ *network in comparison with that from the HIV-RT/DNA-DNA/TFV-DP crystal structure.* **a** The HBV-RT4/DNA-RNA/ *TFV-DP* structure model is shown in cartoon with each domain in different color (N-terminal: orange; fingers: purple; thumb: red; palm: green; dsDNA: silver). The substrate TFV-DP and the residues involved in direct interaction with it are in stick and ball representation; **b** The active site configuration of the HBV-RT4/DNA-RNA/TFV-DP model. The non-bonded interactions are explicitly represented as black dashed lines as the two H-bonds (Hb1 and Hb2), and the ionic bonds formed between the Mg^2+^ ions (in red ball representation without labeling) and the chelating oxygen atoms. The water oxygen atoms participating in the chelation are labeled as from W1 to W4; **c** The active site configuration of the HIV-RT/DNA-DNA/TFV-DP crystal structure, only one Mg^2+^ is identified in the active site and no water molecule is involved. The hydrogen atoms are hided in the representations for clearance

Here we present the HBV-RT4/DNA-RNA/TFV-DP model. As seen in Fig. 4a, the incorporation of the TFV-DP did not elicit any disruption of the secondary structural elements of the HBV-RT4. The domain arrangement persists as they are in the HBV-RT4/DNA-RNA/dATP complex (Fig. 2a), indicating the model is in a proper functional state. At the active site, the TFV-DP associates with the enzyme in the exact fashion as we identified in the natural substrate-bound complex: the base-ring end of the TFV-DP is anchored by two persistent H-bonds, and the triphosphate end of it is strongly coupled by the Mg^2+^ chelation network (Fig. 4b). While Mg^2+^-2 was missing in the HIV-RT/DNA-DNA/TFV-DP crystal structure (Fig. 4c), we believe that the integrity of the Mg^2+^ network is important for maintaining the proper configuration of the active site, as is seen in the HIV-RT/DNA-RNA/dATP crystal structure and in all of our HBV-RT/DNA-RNA/dATP systems with two Mg^2+^ ions. Convinced of the potential catalytic role of the Mg^2+^-2, we opted to include the Mg^2+^-2 in all of our HBV-RT/DNA-DNA/TFV-DP systems. The result that the Mg^2+^ network is stable across all variants echoes the importance and validity of the addition.

We were then able to compute the binding energies of the dATP and TFV-DP to the HBV-RT/DNA-RNA complex with extensive sampling of the complex structures through MD simulations using the solvated interaction energies (SIE) method [24, 25]. The favorable and consistent binding energies of dATP to the variants observed in both Mg^2+^ systems (Table 1) indicate strong binding of the natural substrate to the enzyme, hence proper configuration of the active sites for all of our HBV-RT variant models. As for the TFV-DP ligand, the SIE results also show favorable binding to all HBV-RT variants (Table 1), with no substantial differences between them (<1 Kcal mol^-1^). These findings suggest that RT enzymatic activity and RT’s affinity to bind TFV-DP are not altered by the amino-acid heterogeneity observed among the HBV-RT variants examined herein, which, taken together with the observed stability of ligand-protein interactions and consistency in the structural configuration of the active site in all HBV-RT/DNA-RNA/TFV-DP complexes, suggest that factors other than a decreased binding affinity of HBV-RT to TFV-DP might be influencing therapy response in patients.

Interestingly, evidence of there being no substantial changes in drug-binding affinity in our HBV-RT/DNA-RNA/TFV-DP complexes (Table 1) is in agreement with our previous study [26]. A potential prediction model of response to TDF revealed that genetic diversity outside the RT domain of polymerase contributed robust predictive features strongly associated to SR/RR characteristics of patients, whereas in RT none were found [26]. Moreover, a switch in high-frequency intra-host HBV variants was observed in patient 4, which did not involve changes in RT. Amino-acid substitution S202P (GenBank reference AY AY233278) in the spacer domain of polymerase presented after 4 weeks of TDF treatment, suggesting a possible association between response to TDF and amino-acid heterogeneity in spacer, a suspected intrinsically disordered protein (IDP) [27]. In fact, there is compelling evidence of the involvement of IDPs in evolution of drug resistance in cancer cells [28, 29] and HBV [27]. Nevertheless, an association between amino-acid substitutions in HBV variants RT1–RT5 and differential response to TDF treatment cannot be dismissed solely based on findings presented herein. Drug-binding affinity may not be the only mechanism involved in response rates or resistance to TDF. Amino-acid substitutions observed in HBV-RT could be affecting some other structure-activity-resistance relationships. In addition, intramolecular conformational changes in RT and intermolecular dynamics of the RT complex provide an alternative mechanism for drug resistance to NNRTIs [30].

## Conclusion

While many drug resistant mutants of HBV-RT have been identified, very little is known about the molecular mechanism driving resistance development. Compared to the massive structural and biochemical studies than have been conducted on HIV-RT drug resistant mutants, HBV-RT has not drawn much attention among structural biologists. However, over 350 million people have developed CHB and it remains a serious, global health problem [9]. Therefore, it is important to unravel the drug resistance associated with anti-viral treatment to HBV infections.

To probe the structural basis for HBV-RT drug resistance, researchers rely on structural modeling approaches using crystal structures of HIV-RT as a starting point [17, 18, 31–33]. As protein structures are dynamic and strongly correlated with its functional states, we devised a novel hybrid approach in which we utilized the most relevant X-ray crystal structures (in terms of its functional state) to model the initial structures of HBV-RT and then extensively sampled the conformational space with experimentally derived constraints (crystal contacts between substrate and protein) to select the final representative models (Additional file 3: Figure S3). Without direct experimentally derived structure data on HBV-RT to test the models, this rigorous approach presumably generates structure models of the highest confidence and relevance to the particular biological function of interest. However, as this approach comprises only physics-based methods, it is advisable to apply other methods to evaluate the resulting model from a different perspective. In this study, we have applied a statistical potential based method (ProSA-Web) [34] to evaluate our model. The resulting Z-score for our HBV-RT4 model confirms good quality in terms of the naturalness defined by sets of X-ray and NMR structures (Additional file 4: Figure S4A), and the local quality plot indicates the majority of the HBV-RT4 model is of high quality measured by a knowledge-based energetic term (Additional file 4: Figure S4B).

Using these models to probe drug-protein interaction, we focused on the TFV-DP binding. The present findings indicate that, despite sequence changes in or around the NRTI binding site, favorable drug-protein interactions persisted. The dynamics of how key molecular groups interact, probed in our studies, reveal new insights into the mechanisms, through which HBV-RT couples to its natural substrate. However, this apparent drug-protein interaction stability leaves us without a good mechanistic rationalization as to why or how HBV-RT amino-acid polymorphisms may contribute to reduced rates of viral clearance responses to TDF treatment, as is often observed in SR patients.

The modeling approach presented in this study can be extended to other drugs and serve as a paradigm to ascertain drug binding and estimate binding free energies. It is important to note that the starting conformation (the binding site and the particular interaction pattern between the drug and protein) would have much effect on the results. Therefore, it is essential to start modeling using all available information for the accurate and reliable prediction of the binding site structure.

## Methods

### Patients and HBV quasispecies (QS)

Five patients from Study GS-US-203-0101, a phase 2 study evaluating TDF and FTC + TDF in treatment naive patients with HBV DNA >1.7 x 107 IU/mL and normal ALT levels for 192 weeks, were selected [35]. 3 patients had a slow response (SR), never achieving HBV DNA <69 IU/mL through 192 weeks of treatment, while 2 patients had a rapid response (RR), achieving HBV DNA <69 IU/mL by Week 96. Patients were matched by HBV DNA, ALT, and HBeAg status at baseline. All patients received TDF monotherapy. Whole-genome sequences of HBV QS were obtained using end-point limiting-dilution real-time PCR coupled with sequencing. Baseline (BL), Week 4 (W4), and Week 40 (W40) time points were evaluated for each patient. Median joining networks (MJN) were performed using Network 4.0 to analyze HBV QS genetic diversity [36]. The high-frequency variants of intra-host HBV QS from each patient were selected to predict the atomic structure of the RT protein domain in HBV polymerase (protein positions 349 – 693, based on GenBank reference sequence AY233278): from SR patients, variants RT1 and RT2 of HBV genotype C (GT C) and RT4 (GT B), and from RR patients, variants RT3 (GT C) and RT5 (GT B).

### Structural modeling of HBV-RT variants

Due to the nonexistence of experimental 3D-structures of HBV-RT, a hybrid approach to protein 3D-strucutre prediction was implemented to generate accurate, atomistic structures of the NRTI binding site of HBV-RT. Three major steps were involved in the modeling. First, all the representative HBV-RT sequences were submitted to ITASSER web-server [20, 21], including specification on an homologous HIV-RT structure complex (PDB accession code: 4PQU) as an additional template [20]. The model of the highest C-score [21] were then chosen to proceed. The N-terminal 1-40 amino-acid of HBV-RT were modeled separately by ITASSER, due to a relatively low local accuracy for the initial model of the entire length. The N-terminal structure was then assembled to the preliminary model by using *Ab Initio* Domain Assembly Server (AIDA) [23].

Second, all the HBV-RT variant models were then pooled for inspection to identify the one(s) with the folding pattern most consistent with that seen in the designated HIV-RT structure.

Last, variant HBV-RT4 was chosen for further refinement using multi-nanosecond molecular dynamics (MD) simulation with the native hybrid DNA-RNA duplex and with dATP incorporated into the active site, according to their original orientation in the HIV-RT/DNA-RNA/dATP crystal structure [22]. Pairwise rmsd clustering analysis of the second half of the MD trajectory was then performed to select the centroid of the dominant cluster as the final model of HBV-RT4/DNA-RNA/dATP. The structures of the other variants were then obtained by homology modeling using Modeller 9.13 [37, 38] with the HBV-RT4 as template_,_ followed by same MD simulation procedures, also with the native hybrid DNA-RNA duplexes and the dATPs superimposed to the active sites.

In the case of modeling the HBV-RT4/DNA-RNA/TFV-DP variants, we replaced the dATP with TFV-DP in the initial conformation of the HBV-RT4/DNA-RNA/dATP MD simulation, maintaining the crystal contacts between TFV-DP and corresponding residues that was identified from the HIV-RT4/DNA-DNA/TFV-DP crystal structure (PDB code: 1 T05) [19].

### Molecular dynamic (MD) simulation

To conduct MD simulations, hydrogens were first added by the tleap module of AMBER 14 [39], and the ionizable side chains of the proteins were assigned to their ionization states at pH 7.0 using the WHATIF webserver [40]. Each system was then solvated with TIP3P water molecules [41] leaving a minimum distance of 10.0 Å from the protein surface to the edge of the simulation box. Counter-ions were added to neutralize the net charge and reach 100 mM NaCl concentration to mimic physiological conditions. The systems were then minimized and equilibrated -- with extra bonds constraints (2.5 kcal mol^−1^ Å^−2^) on the Mg^2+^ ions and on the ionic bonded oxygen atoms -- for 10–20 ns before free production runs in the isothermal isobaric ensemble (1 atm, 300 K). The short-range non-bonded interactions were evaluated by employing a cut off of 10 Å with a switching function starting at 8.5 Å. The smooth particle mesh Ewald (SPME) algorithm were applied in computing the ong-range electrostatic interactions [42]. All the bonds formed by hydrogen and heavy atoms were fixed to eliminate the most frequent oscillatory motions. The r-RESPA multiple time step method [43] was applied with a 2 fs time step for bonded, 2 fs for short-range non-bonded interactions and 4 fs for long-range electrostatic interactions. All simulations were performed using the NAMD 2.9 code [44, 45] with the AMBER Parm99 parameter set [46] containing the force field for nucleic acids and proteins. The parameterization of TFV-DP was conducted using Antechamber [47] with the general Amber force field (GAFF) [46, 48]. Data were analyzed using the CPPTRAJ utility in AMBER [49] and custom VMD TCL scripts [50]. Time evolution of the rmsd values for the HBV-RT in each simulation system was monitored for simulation convergence (Additional file 5: Figure S5).

### Binding free energy approximation

To compute the binding free energy of dATP or TFV-DP to the HBV-RT/DNA-RNA complex we employed the solvated interaction energies (SIE) method [24, 25], which is an end point method that shares elements from the linear interaction energy (LIE) approach [24, 25]. Frames at 20 ps intervals from the last 60 ns the MD trajectories (the rest of the trajectories were discarded as equilibration) were sampled for the SIE calculations. In total 3000 frames were used for averaging. The SIE estimates binding free energy as:

$$ \varDelta {G}_{bind}\left(\rho, {D}_{in},\alpha, \gamma, C\right)=\alpha \times \left[\varDelta {E}_{vdW}+\varDelta {E}_{Coul}\left({D}_{in}\right)+\varDelta {G}_{RF}\left(\rho, {D}_{in}\right)+\gamma \varDelta SA\left(\rho \right)\right]+C $$

where *ΔE*_*vdW*_ and *ΔE*_*Coul*_ represents the intermolecular van der Waals and Coulomb interaction energy between protein and ligand, respectively. The values were computed using he AMBER ff99SB parameter set. *ΔG*_*RF*_ is the reaction field energy change upon ligand binding, computed by solving the Poisson equation with program BRI BEM [51], and using a molecular surface generated with a variable-radius solvent probe [52]. The *ΔSA* is the molecular surface area change upon ligand binding. *ρ* is the linear scaling factor of the van der Waals radii for the AMBER99 force field; *D*_*in*_ is the dielectric constant for solute interior; *γ* is the coefficient for computing the free energy associated with the surface area change upon binding; *α* is the pre-factor that quantifies the loss of configurationally entropy upon binding; *C* is a constant that includes protein-dependent contributions not explicitly modeled by SIE. The parameters were fitted to the absolute binding free energies for a set of 99 protein-ligand complexes to obtain the optimal/default values of them as

*α* = 0.1048, *D*_*in*_ = 2.25, *ρ* = 1.1, *γ* = 0.0129 Kcal mol^− 1^Å^− 2^, and *C* = − 2.89 Kcal mol^− 1^ [24, 25], which are also the values we used in our SIE calculations.

## Abbreviations

CHB, chronic hepatitis B; dATP, deoxyadenosine triphosphate; DNA, deoxyribonucleic acid; ds-DNA, double stranded DNA; HBV, hepatitis B virus; MD, molecular dynamics; NNRTI, non-nucleoside reverse transcriptase inhibitors; NRTIs, nucleoside and nucleotide reverse transcriptase inhibitors; rmsd, root mean square deviation; RNA, ribonucleic acid; RR, rapid response; RT, reverse transcriptase; SIE, solvated interaction energy; SR, slow response; TDF, tenofovir disoproxil fumarate; TFV-DP, tenofovir diphosphate (active form of TDF).

## Additional files

Additional file 1: Figure S1.
*The initial structural models of HBV-RT variants obtained from ITASSER in comparison to the corresponding part in the template crystal structure*: A) HBV-RT5; B) HBV-RT1; C) HBV-RT4; D) HBV-RT2; E) HBV-RT3; F) Residues 1 to 320 of the HIV-RT/DNA-RNA/dATP crystal structure (PDB code: 4PQU). The 40 aa N-terminal of the HBV-RT structure are shown in orange, the hairpin structural element in the fingers domain are in red, and the rest of the structure are in cyan. (TIF 5479 kb) Additional file 2: Figure S2.
*The structure models of HBV-RT4 obtained from ITASSER followed by ab initio assembly using AIDA.* The 40 aa N-terminal of the HBV-RT structure is shown in orange, the hairpin structural element in the fingers domain in red, and the rest of the structure in cyan. (TIF 5196 kb) Additional file 3: Figure S3. The schematic plot of the hybrid modeling approach used in HBV-RT/DNA-RNA/dATP and in HBV-RT/DNA-RNA/TFV-DP modeling. (TIF 2413 kb) Additional file 4: Figure S4.
*Quality of HBV-RT4 model evaluated by statistical potential:* A) Overall quality score of the HBV-RT4 model (black dot): Z-score =−6.34; B) Local model quality scores as knowledge-based energies versus residue position, with positive values indicate possible problematic or erroneous parts in the model. (TIF 2605 kb) Additional file 5: Figure S5.
*RMSD evolution as a function of time for all the MD simulations:* A) HBV-RT/DNA-RNA/dATP with Mg^2+^-1 only in the active site; B) HBV-RT/DNA-RNA/dATP with two Mg^2+^ ions in the active site; C) HBV-RT/DNA-RNA/TFV-DP with two Mg^2+^ ions in the active site. (TIF 2569 kb)
